# Lipid accumulation in adipose tissue-resident iNKT cells contributes to an inflammatory phenotype

**DOI:** 10.1080/21623945.2024.2421750

**Published:** 2024-11-01

**Authors:** Imogen Morris, Frank Vrieling, Annemieke Bouwman, Rinke Stienstra, Eric Kalkhoven

**Affiliations:** a Ce nter for Molecular Medicine, University Medical Center Utrecht, Utrecht University, Utrecht, The Netherlands; b Nutrition, Metabolism and Genomics Group, Division of Human Nutrition and Health, Wageningen University, Wageningen, The Netherlands; c Department of Internal Medicine, Radboud University Medical Center, Nijmegen, The Netherlands

**Keywords:** iNKT cell, adipose tissue, lipids, lipid droplet, inflammation

## Abstract

Reciprocal communication between adipocytes and immune cells is essential to maintain optimal adipose tissue (AT) functionality. Amongst others, adipocytes directly interact with invariant NKT cells (iNKT cells), which in turn secrete various cytokines. A lipid-rich microenvironment, as observed in obesity, skews this adipocyte-driven cytokine output towards a more inflammatory output. Whether a lipid-rich microenvironment also affects iNKT cells directly, however, is unknown. Here, we show that primary mouse iNKT cells isolated from AT can accumulate lipids in lipid droplets (LDs), more so than liver- and spleen-resident iNKT cells. Furthermore, a lipid-rich microenvironment increased the production of the proinflammatory cytokine IFNγ. Next, to an indirect, adipocyte-mediated cue, iNKT cells can directly respond to environmental lipid changes, supporting a potential role as nutrient sensors.

## Introduction

Metabolism and immunology interact at numerous levels, leading to the emergence of the novel field of immunometabolism [1]. An important immunometabolic interface is formed by cell–cell communication between adipocytes and immune cells take occurs in adipose tissue (AT). One side of the immunometabolic interface is formed by adipocytes, a highly specialized cell type characterized by the ability to store lipids as triglycerides in so-called lipid droplets (LDs). During times of increased energy demand, lipolysis hydrolyses the TG stored in the LDs back into FFAs that are subsequently released by the adipocytes. LDs are very common in all eukaryotes and can be found in many organelles, including the endoplasmic reticulum (ER), Golgi apparatus, lysosomes, secretory vesicles and others [1]. The ER and Golgi have been long-recognized as mammalian cells’ main site of LD processing [2]. LDs have a highly conserved structure and proteome and are present in many organisms, from bacteria to algae and plants, insects to humans [2]. It has even been suggested that LDs are so highly conserved and ubiquitous that they could be one of the most ancient organelles [3,4]. LDs are comprised of a core of neutral lipids surrounded by a phospholipid monolayer and associated proteins (class 1 and 2). These small organelles have been shown to have a highly dynamic life cycle, providing protection from lipotoxicity and enabling cells, including adipocytes, to function, grow and divide (reviewed [5,6]).

Interestingly, adipocytes share some phenotypic aspects of immune cells: adipocytes express the machinery for both peptide antigen and lipid antigen presentation, they have secretory capacities (cytokines and adipokines), and the adipocyte gene expression profile is regulated by transcription factors like PPARs and LXRs, which also play an essential role in various immune cells [7,8]. On the other hand, immune cells, which form the other side of the immunometabolic interface, share features with adipocytes. One example of this is the storage of triglycerides in LDs, first reported in macrophages/monocytes, dendritic cells and lymphocytes [9]. Catabolism and storage of lipids in these immune cells have been shown to impact their functional phenotype [10,11]. For instance, excessive storage of lipids in macrophages can lead to a pro-inflammatory phenotype and promote pro-inflammatory cytokine secretion, which can further recruit more immune cells to the inflammatory sites [12,13].

One particular type of immune cells, invariant Natural Killer T-cells (iNKT cells), has attracted attention in obesity for several reasons: (i) in healthy AT they can make up to 20% of the immune cell population [14,15], (ii) AT-resident iNKT cell numbers decline in obesity and various mouse models indicate that they support optimal AT function [14,16], (iii) iNKT cells can be activated by lipid antigens presented in the context of CD1d by adipocytes [16–18]. While iNKT cells can secrete both anti- and pro-inflammatory cytokines and thereby regulate other AT-resident immune cell types, they predominantly secrete anti-inflammatory cytokines in healthy AT [19] and can be skewed towards a more pro-inflammatory phenotype under obese conditions, in vitro [18]. Furthermore, the overall phenotype of iNKT cells can vary substantially by the tissue they reside in (e.g. AT vs liver), but if and how the lipid-rich environment of AT directly influences iNKT cells is largely unexplored. Here, we examined the effects of a lipid rich environment on iNKT cells isolated from different tissues (AT, liver, spleen) in vitro and report their ability to store lipids in LDs. Lipid storage resulted in the secretion of higher levels of the pro-inflammatory cytokine IFNγ, most pronounced in AT-resident iNKT cells. We conclude, therefore, that next to an indirect, adipocyte-mediated cue, iNKT cells can directly respond to environmental lipid changes, supporting a potential role as nutrient sensors.

## Results

### The iNKT hybridoma cell line DN32.D3 can store environmental lipids as lipid droplets

**Figure 1.**
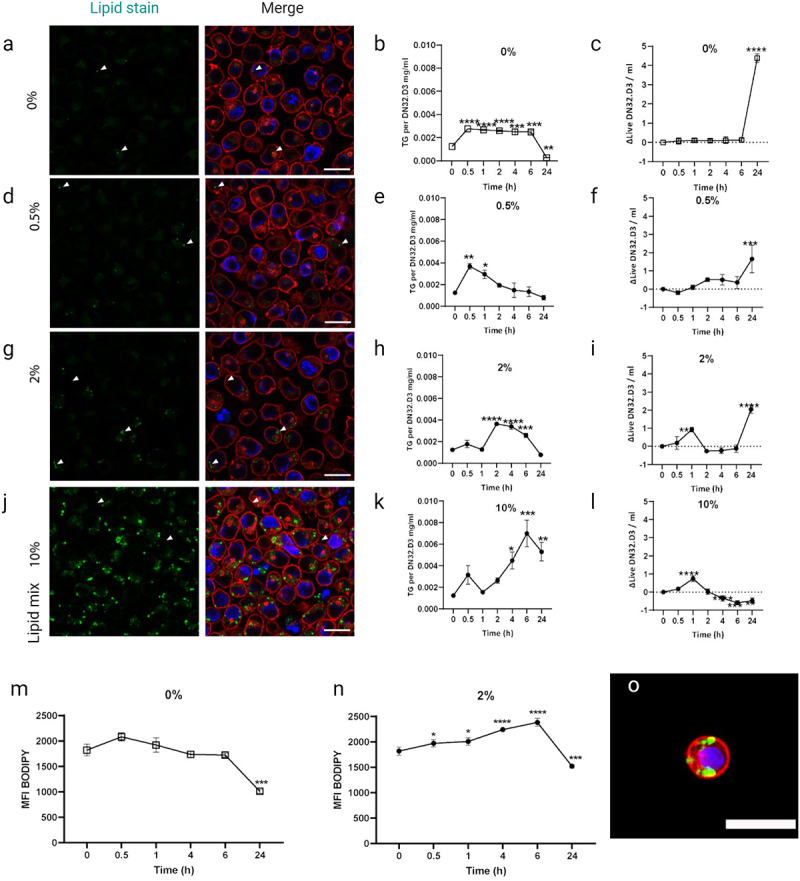
DN32.D3 iNKT cell line loads and stores environmental lipids as lipid droplets in a dose dependent manner.

To investigate the potential lipid loading capacity of iNKT cells and subsequent effects on their phenotype, we first developed a protocol based on previous work [18,20] where we exposed mature 3T3-L1 adipocytes to a mix of exogenous lipids, including different FFAs via media conditioning. To allow extensive optimization in these initial stages, we used the murine iNKT cell hybridoma line DN32.D3 [21], which has served as an iNKT cell model in various studies [18,22,23]. To visualize lipid accumulation, cells were stained for neutral lipids with BODIPY and analysed with confocal microscopy, and F-actin and DNA were stained with Phalloidin and DAPI, respectively, to identify individual cells. In parallel, lipid accumulation was quantified by colorimetry, and cell viability was assessed. Using standard growth media as control condition, we consistently observed a few lipid droplets in a subset of DN32.D3 cells, suggesting that these cells harbour the intracellular pathways required for proper LD formation (Figure 1a,b). To simulate an *in vivo* high lipid environment, cells were incubated with 0.5%, 2% and 10% of a defined lipid mixture, composed of fatty acids and cholesterol (2 μg/ml arachidonic acid and 10 μg/ml each linoleic, linolenic, myristic, oleic, palmitic, stearic acid and 0.22 mg/ml cholesterol), for 12 h showed a dose-dependent increase in the amount of lipid droplets (Figure 1a-l). To assess how quickly lipids accumulate in this cell system, we incubated with lipid mix for different time periods (0.5 h–24 h), and elevated TG content was already observed after 0.5 h–2 h with all concentrations of lipid mix (Figure 1b,e,h,k). In the control setting, TG uptake remained stable until 24 h, where we observed a significant decrease in TG content (Figure 1b) accompanied by increased proliferation (Figure 1c). Upon incubation with 0.5% and 2% lipid mix, the initial TG accumulation was accompanied by a modest increase in viability, especially at 24 h (Figure 1f,i). Incubation with 10% lipid mix gave the highest level of TG accumulation (Figure 1k) but at the same time reduced cell proliferation (Figure 1l). Taken together, these results show that exposure to exogenous lipids rapidly results in TG accumulation in DN32.D3 over a range of concentrations, with prolonged incubations at high concentrations resulting in reduced cell viability. Based on these findings, subsequent experiments were performed with 1–2% lipid mix for 1 h (Supplemental Figure S1). Finally, we tested flow cytometry as a method to assess immune cell populations for the presence of lipid droplets using BODIPY or LipidTox. Flow cytometry data (Figure 1m,n) concurred with our imaging and TG quantification (Figure 1b,h), showing this to be a reliable additional readout method. We also stained and imaged iNKT cells extracted from visceral AT (Figure 1o), we found visible lipid droplets confirming this phenomenon within this immune cell population.

(a) DN32.D3 cells cultured in a growth medium for 12 h stained for lipids (Bodipy in green), DNA (DAPI in blue) and actin (Phalloidin in red). (b) Intracellular triglyceride content of DN32.D3 (mg/ml). (c) Delta change of live dead ratio of DN32.D3 over a 24-h period of culture. (d–f) 0.5% Lipid mix. (g–i) 2% Lipid mix. (j–l) 10% Lipid mix. (m) 24-h culture of DN32.D3 were cultured in growth media (n), or 2% lipid mix over a 24-h period, stained for lipid content via Bodipy then analysed for mean fluorescent intensity (MFI) by flow cytometry (10.000 events/run). (o) Lean visceral AT-resident iNKT cells stained as DN32.D3 above. (ns *p* > 0.05, *p < 0.05, **p < 0.01, ***p < 0.001, ****p < 0.0001), white scale bar is 20 µm, *n* = 3.

### AT-resident iNKT cells store lipids and display lipid-induced IFNγ production

**Figure 2.**
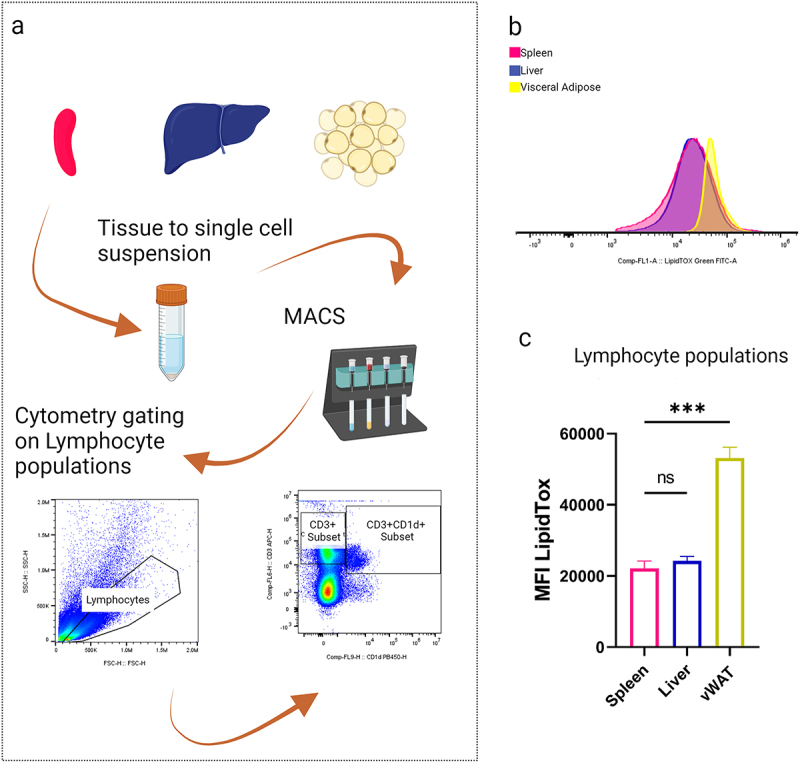
*Ex-vivo* gated lymphocytes show a lipid loaded phenotype.

Having observed that the iNKT hybridoma cell line DN32.D3 can store exogenous lipids in LDs (Figure 1), we next wished to investigate this in primary mouse iNKT cells. As the phenotype of iNKT cells depends on the tissue they reside in [18,24,25], we set out to compare iNKT cells from spleen, liver and AT. As iNKT cells are part of the lymphocyte population, which includes many immune cell types whose LD content has been shown to modulate their cytokine output [26–28], we first examined the lymphocyte population as a whole and subsequently CD3+ lymphocytes before focusing on tissue-resident iNKT cells (Figure 2a and Supplemental Figure S2).

(a) Representative histogram of lipid stained lymphocyte populations from spleen, liver and vWAT. (b) MFI of lipid stained lymphocyte populations (ns *p* > 0.05, *p < 0.05, **p < 0.01, ***p < 0.001, ****p < 0.0001), *n* = 3.

Lymphocytes from spleen, liver and AT were found to contain lipids (Figure 2b), with lymphocytes extracted from AT containing significantly more lipids than either spleen or liver lymphocytes (Figure 2c). These findings support the view that lipid droplets are a generic organelle present in many immune cell populations and that immune cells residing in higher lipid environments, i.e. AT, are adapted to import and store higher concentrations of environmental lipids.

### AT-resident CD3+ cells specifically produce more IFNγ upon lipid loading

**Figure 3.**
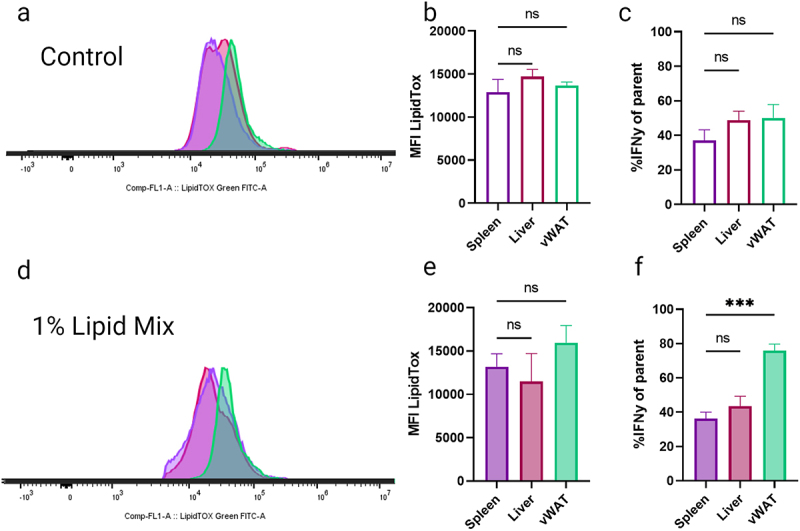
*Ex-vivo* gated CD3+ cells do not load lipids in an organ dependent phenotype.

Before assessing the specifically marked iNKT population, we took advantage of general immune cell population markers, such as CD3, to gauge the effects of various lipid environments on comparable populations. We examined CD3+ cells, a diverse branch of lymphocytes with distinct functions, including T cells. Previously, the composition of their lipid environment has been reported to heavily influence the metabolism of extracellular lipids and the IFNγ response of CD3+ cells [28], which prompted us to focus on this cytokine. IFNγ is also a key cytokine secreted by iNKT cells in various biological settings [19,29] and has been associated with insulin resistance in cultured adipocytes [18,30,31] and in vivo [32,33]. In contrast to the general lymphocyte populations (Figure 2), we observed no significant difference in lipid content between CD3+ cells from different tissues (Figure 3a-b). Nonetheless, CD3+ cells from liver and AT contained a significantly higher proportion of IFNγ producing cells (Figure 3c). Furthermore, when exposing these cells to exogenous lipids, again no clear differences in lipid accumulation were observed between the tissues (Figure 3d-e), but CD3+ cells from AT responded significantly more pronounced with respect to IFNγ production (Figure 3f).

(a) Representative histogram of lipid stained *ex-vivo* CD3+ populations from spleen, liver and vWAT. (b) MFI of lipid stained CD3+ populations. (c) CD3+ cells %IFNγ of parent populations. (d) Representative histogram of *ex-vivo* CD3+ populations conditioned for 1 hour in 1% lipid mix spiked growth media. (e) MFI of lipid stained CD3+ populations. (f) CD3+ cells %IFNγ of parent populations. (ns *p* > 0.05, *p < 0.05, **p < 0.01, ***p < 0.001, ****p < 0.0001), *n* = 3.

### AT-resident iNKTs contain elevated lipid levels and respond rapidly to high lipid environments

**Figure 4.**
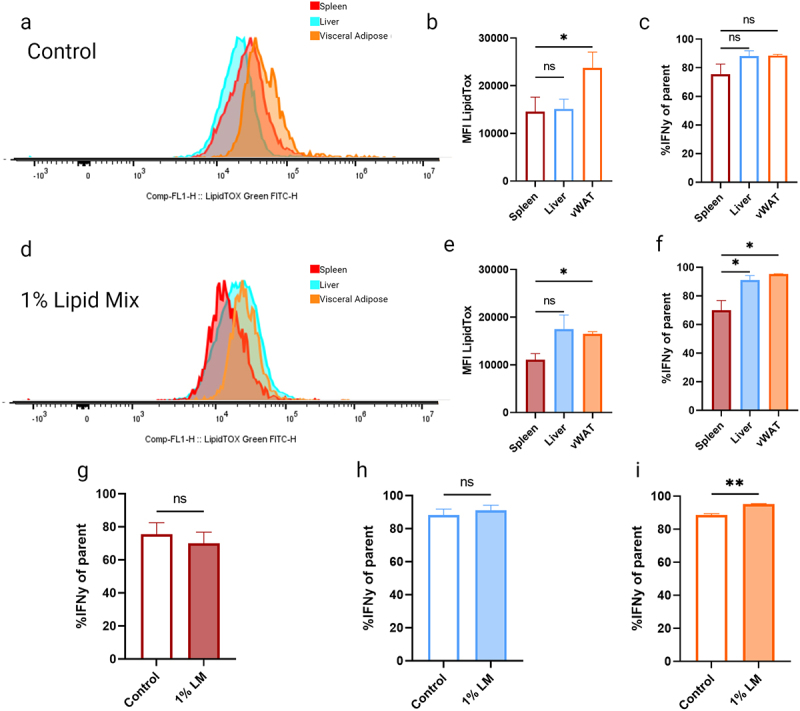
iNKT populations conditioned in 1% lipid mix significantly elevate their IFNγ in an AT-specific manner.

Having observed phenotypic differences between immune cell populations from different tissues (Figures 2 and 3), we finally examined the role of tissue environment in lipid storage and IFNγ production [25,34,35] of iNKT cells. We therefore gated the immune cells further using the iNKT cell-specific glycolipid loaded CD1d tetramer [36,37]. Lipid staining revealed that AT-resident (CD3+CD1d+) iNKT cells contain significantly higher lipid levels when compared to cells isolated from spleen and liver (Figure 4a,b) but share a similar percentage of IFNγ+ cells when compared to liver-resident iNKT cells (Figure 4c). After 1 h of 1% lipid mix conditioning, both liver-resident and AT-resident iNKT cells contained significantly more lipids than spleen-resident iNKT cells (Figure 4d,e), with nearly all iNKT cells now producing IFNγ (Figure 4f-i). Additionally, we assessed CD44 expression, as this is a maturation marker in iNKT cells with higher levels linked to higher IFNγ production [24]. We found no significant changes in CD44+ low or high subpopulations upon 1% lipid mix exposure (Supplemental Figure S3a-b), indicating that the lipid mix-induced increase in IFNγ production is not an indirect effect of iNKT cell maturation.

(a) Representative histogram of lipid stained *ex-vivo* CD3+CD1d+ (iNKT) populations from spleen, liver and vWAT. (b) MFI of lipid stained CD3+CD1d+ (iNKT) populations. (c) CD3+CD1d+ (iNKT) cells %IFNγ of parent populations producing the cytokine IFNγ. (d) Representative histogram of *ex-vivo* CD3+CD1d+ (iNKT) populations conditioned for 1 h in 1% lipid mix spiked growth media. (e) MFI of lipid-stained populations. (f) %IFNγ of population following a 1% LM incubation. (g) Spleen %IFNγ control vs 1% LM. (h) Liver %IFNγ control vs 1% LM (i) vWAT %IFNγ control vs 1% LM (ns *p* > 0.05, *p < 0.05, **p < 0.01, ***p < 0.001, ****p < 0.0001), *n* = 3.

Taken together, these data show that AT-resident iNKT cells display a distinct tissue-specific phenotype, including high lipid storage under basal conditions and increased IFNγ production upon exposure to exogenous lipids.

## Discussion

Our study demonstrates that various iNKT cell populations can uptake environmental lipids, storing them in a tissue-specific manner (Figure 4). iNKT cells immunometabolism regulation relies heavily on their specialization to lipid environment (reviewed [29,38,39]). We propose that iNKT cells, and particularly the AT-resident population, are highly sensitive to lipid environments and can more rapidly take up and store environmental lipids when compared to other immune cell populations (Figures 2 and 3). Furthermore when compared to CD3+ immune populations, comprised of many immune cell types (Figure 3), AT-resident iNKT cells, which are pre-adapted to a high lipid environment, show heightened responsiveness to changes in environmental lipids (Figure 4), setting them apart from other iNKT populations in an AT-specific manner. Remarkably, we noticed that CD3+ cell populations did not show clear tissue-specific differences in lipid content or lipid loading, in contrast to the total lymphocyte populations and iNKT cell populations (Figures 2–4). While further investigations are required, it is possible that potential differences between the CD3+ cell populations are somehow masked, e.g. through opposite lipid loading phenotypes being present within the cell populations.

Adipocytes can tolerate high levels of environmental FFAs as well as sustained uptake and storage during obesity. Our understanding of AT has drastically changed in the last decade, transforming from an inert storage place to a highly dynamic vascularized, innervated hub where metabolism and immunity intersect [40–44]. In AT, immunometabolism plays a key role in local and global homoeostasis, facilitated by various types of co-stimulation between the adipocyte and immune cell [27,45]. Co-stimulation through CD40/CD40L interaction or cytokines can contribute to iNKT cell activation [29], for which CD1d-mediated lipid antigen presentation remains the primary driving force. However, even in the absence of CD1d-mediated lipid antigen presentation, external stimuli can directly influence iNKT cell phenotypes [29]. AT-resident iNKT cells exhibit a specific requirement for AMPK and fatty acid metabolism, linking nutrient sensing to immune function. AMPK, a nutrient sensor, plays a pivotal role in regulating glucose and fatty acid uptake, with consequences for lipogenesis, lipolysis and fatty acid oxidation [46].

Like adipocytes, many other cell types can take up environmental lipids and store them in droplets, enabling rapid nutrient supply and a buffer for lipotoxicity (reviewed [5]). AT-resident iNKT cells, show heightened responsiveness to changes in the environmental lipidome, indeed their journey to AT residency is marked by their loss of the common iNKT transcription factor PLZF, setting them apart from other tissue resident iNKT populations [47]. LaMarche et al. previously showed splenic iNKT uptake of palmitic acid, elevated LipidTox signal, resulted in upregulation of E4BP4 and downregulation of PLZF [25]. AT-resident iNKT cells, being more sensitive to lipid uptake, particularly free fatty acids (FFAs), exhibit a distinct cytokine response profile compared to other resident iNKT populations [19]. This sensitivity is further underscored by the observed alterations in iNKT cell phenotype during the weight gain/iNKT decline phase of obesity, transitioning from anti-inflammatory to pro-inflammatory states [14,18,25,48]. iNKT cells regulation of local immune cells and their sensitivity to environmental changes have put them at the forefront of various research topics, ranging from cancer to diabetes (reviewed but not limited to: [38,49–52]). While much progress has been made in understanding the evolution and conservation of lipid droplets (LDs) in various species and cell types, the role of LDs in immune cells and inflammation remains an area of active investigation. Here, we highlight the importance of LDs in modulating immune responses and inflammation, suggesting that LDs may be a potential target for therapeutic intervention.

## Materials and methods

### Cell culture and lipid loading assays

The murine iNKT hybridoma cell line DN32.D3, kindly donated by the Brennan lab, was cultured in Roswell Park Memorial Institute (RPMI) medium (Sigma) supplemented with 10% foetal bovine serum (Bodinco BV), 1% Penicillin–Streptomycin (Sigma), 1% L-Glutamine (Sigma), 2% HEPES (Gibco) and beta-mercapto-ethanol (100 μM). Media was filtered before use. DN32.D3 were grown in suspension flasks up to a confluence of 1 × 10^6^ cells/mL, and the live/dead ratio was maintained at 95–99% live as evaluated using the Countess II Automated Cell Counter (Invitrogen). Lipid loading was performed by adding Lipid Mixture 1, Chemically Defined (Sigma, L0288) to a regular medium. Before experiments, DN32.D3 was counted, centrifuged (300 G for 5 min) and resuspended in media containing lipid mix at a confluence of 2–3×10^5^ cells/mL. After indicated time points, cells were washed in PBS, fixed in 4% PFA for 30 min at room temperature, washed twice in PBS and stored at 4°C until further analysis. The HeLa: CD1d cell line was cultured in Dulbecco’s Modified Eagle’s Medium (DMEM) – high glucose (Sigma) supplemented with 10% FBS (Bodinco BV) and 1% Penicillin–Streptomycin (Sigma).

### Lipid staining

Fixed DN32.D3 were stained for neutral lipids using HCS LipidTox Green Neutral Lipid Stain (1:1000, Invitrogen, H34475) or BODPIY 493/503 dye (1:1500, Invitrogen, D3922) for 30 min at room temperature. Acquisition was done within 2 h using a FACSCelesta (BD) 488 laser with a BB515 filter using FACSDiva Software (BD Life Sciences). Flow cytometry results were analysed using FlowJo v10.8 Software (BD Life Sciences).

### Triglyceride measurements

Intracellular triglycerides were measured using the kit Stanbio™ Triglycerides LiquiColor™ (#SB2200-225). Cells were washed with PBS and lysed in 50 μl cold PBS via syringe pull technique, then analysed as per manufacturer’s instructions.

### Confocal microscopy

Samples were stained with Hoechst 3342 (1:1000), BODPIY 493/503 dye (1:500, Invitrogen, D3922) and Phalloidin 647 (1:1000). Within 2 h, samples were imaged on a Zeiss laser scanning microscope (LSM)880, using a 40× water immersion objective. Brightness and contrast were adjusted using Fiji version 1.52 g. (National Institutes of Health, USA).

### Flow cytometry and analysis

Spleen, liver and visceral AT were collected and pooled from four standard chow fed male C57BL/6J mice of 9–11 weeks old. Spleens and livers were dissociated through a 70 μm cell strainer into 50 mL ice-cold PBS and centrifuged at 400 G for 10 min. Red blood cells were lysed in 5 mL Red Blood Cell Lysis Buffer for 5 min at room temperature, washed in PBS, and filtered through a 70 μm filter. Visceral AT was minced after removal of blood and lymph nodes and kept in ice-cold digestion buffer (Hanks’ balanced salt solution (HBSS) with Ca^2+^ and Mg^2+^ supplemented with 0.5% bovine serum albumin). Per 1 g AT, 1 ml of 10 mg/ml collagenase (Sigma-Aldrich; C6885) was added and incubated at 37°C for 15–20 min with vortexing every 10 min until a single cell suspension was achieved. AT single cells were then passed through a 100 μm filter. iNKT cells were isolated from the single-cell suspensions using the NK1.1^+^ iNKT Cell Isolation Kit (Miltenyi Biotec, 130–0960513) in which the Anti-NK1.1-APC antibody was replaced for mCD1d tetramer-APC (NIH Tetramer Core Facility). NK1.1 kit cocktail, CD115, CD8a, CD45R, Nkp46 (CD33) and TCR (gamma lambda yd). The single cell suspensions were then resuspended in 100 µl PBS incubated with PMA (BD 5 ng/ml Sigma P8139) and ionomycin (500 ng/ml Sigma I0634) combined with GolgiStop (BD 554,724), followed by the FIX & Stanbio™ Cell Permeabilization Kit (GAS003), with the antibodies, CD3 (BioLegend 100,203), CD44 (BioLegend 103,031), IFNγ (BioLegend 502,527), OHC loaded tetramer CD1d (NIH Tetramer Core Facility) and LIVE/LiquiColor™ Fixable Aqua Dead Cell Stain Kit (PERM™ L34957) on ice. Cells were washed twice with staining buffer, resuspended in 200 µl staining buffer and analysed within 1 h using a CytoFLEX S cytometer (Beckman Coulter).

## Supplementary Material

Supplemental Material

## Data Availability

All datasets generated for this study are publicly available at https://zenodo.org/records/11472061 and https://zenodo.org/records/12546955.

## Data representation and statistics

Flow cytometry data were analysed using FlowJo software version 10.8.1 (Becton Dickinson). All graphs and statistical analyses were generated using GraphPad Prism 9.3.1. Brown-Forsythe and Welch One-Way ANOVA, with Dunnett’s T3 multiple comparison tests.
