# Supplementary figures and images for: Lipid accumulation in adipose tissue-resident iNKT cells contributes to an inflammatory phenotype
Source: Adipocyte. 2024 Nov 1;13(1):2421750. doi: 10.1080/21623945.2024.2421750 (PMC11540091; doi:10.1080/21623945.2024.2421750)

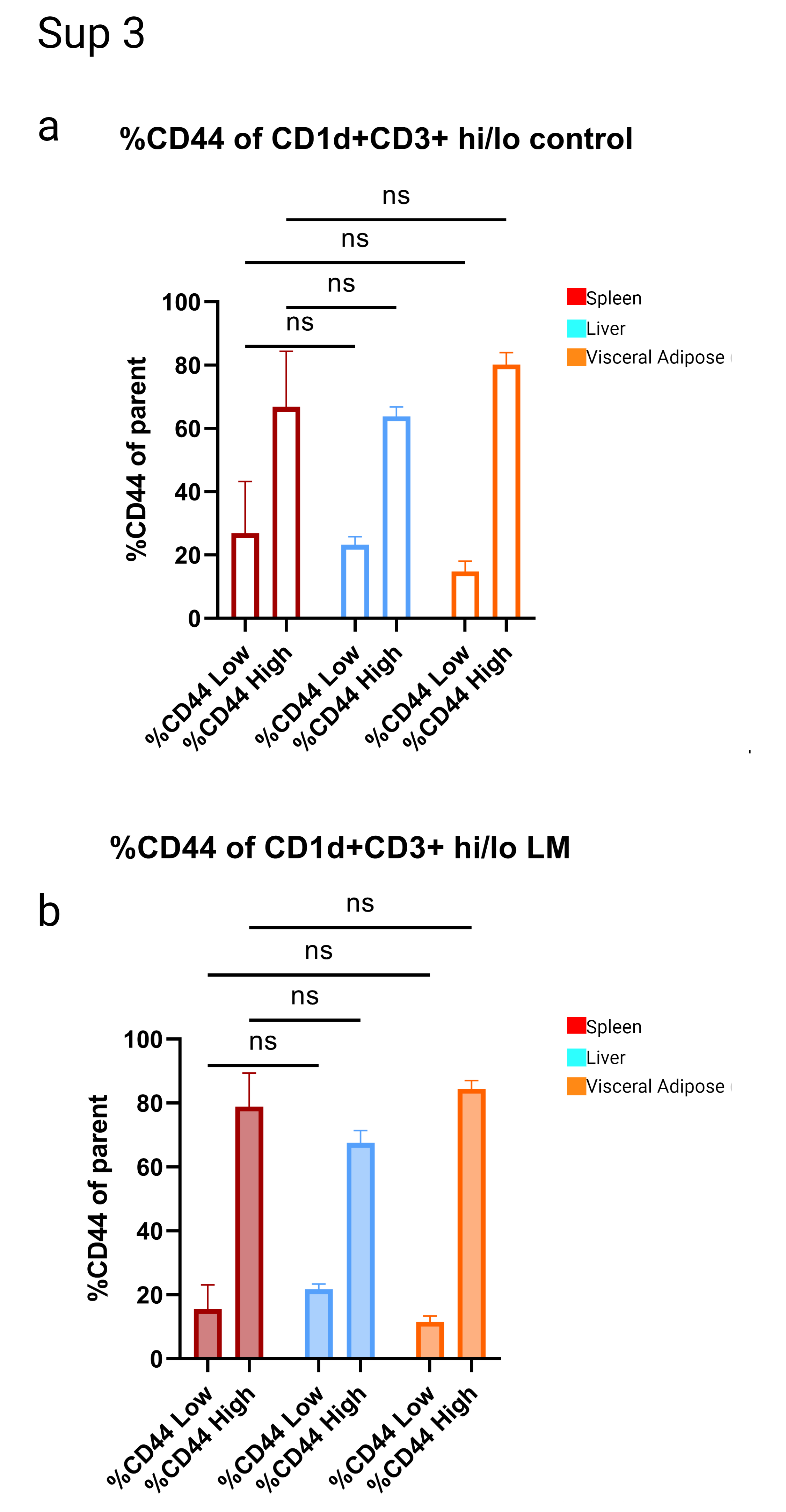

Supplement: Supplemental Material [file KADI_A_2421750_SM1643.zip › S3 .jpg]

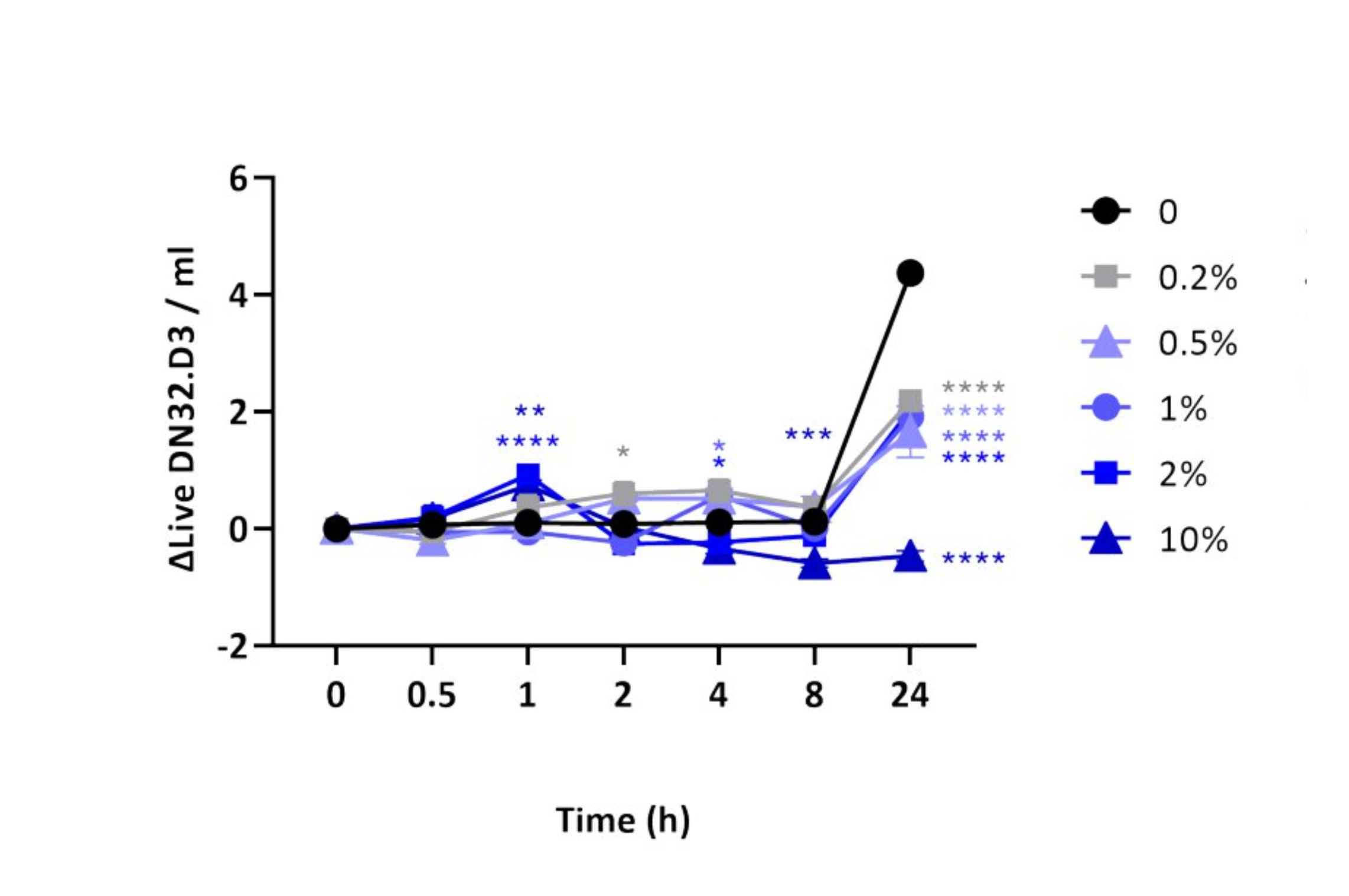

Supplement: Supplemental Material [file KADI_A_2421750_SM1643.zip › Sup1.png]

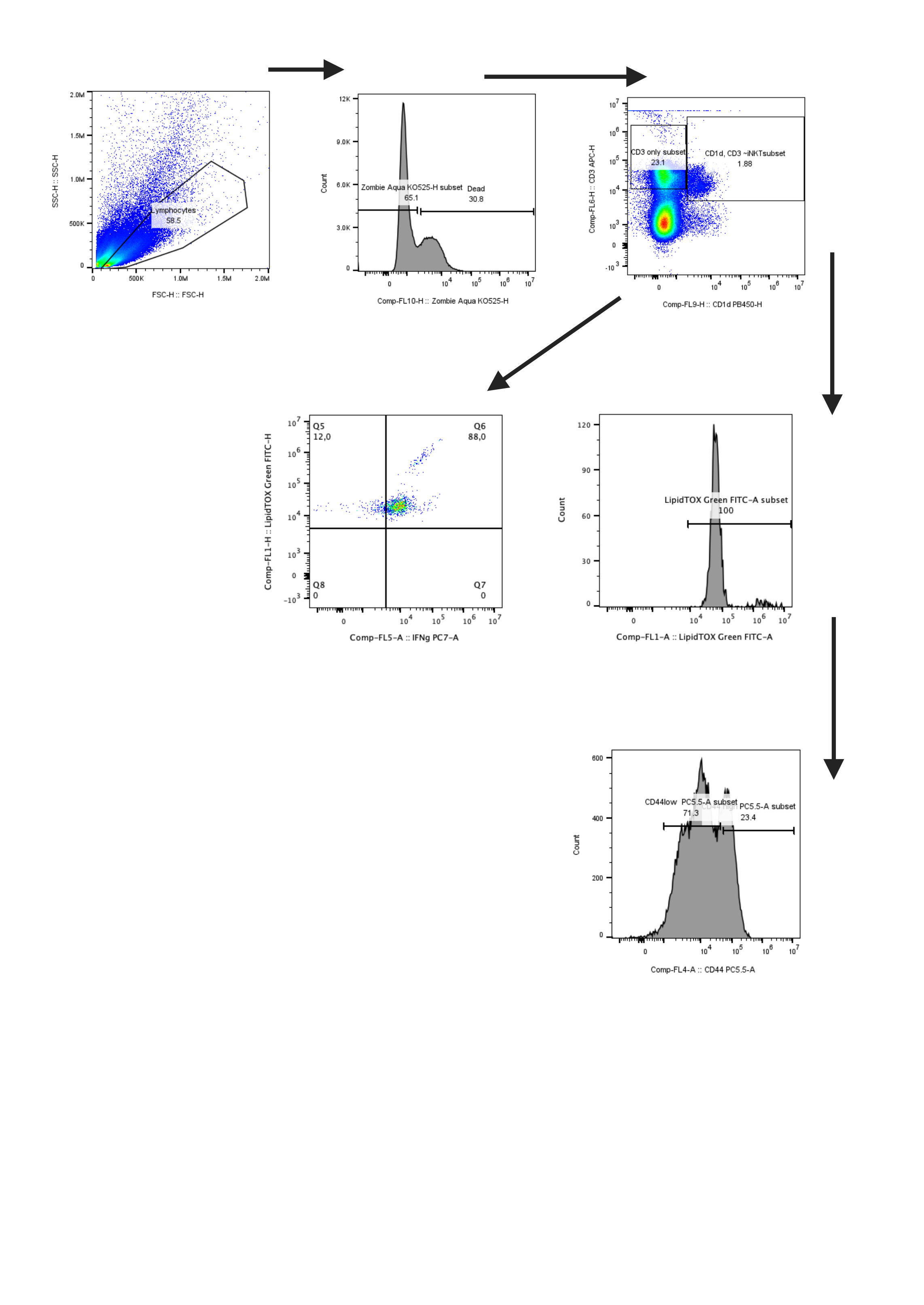

Supplement: Supplemental Material [file KADI_A_2421750_SM1643.zip › Sup2.png]
